# Radiation Measurements Performed with Active Detectors Relevant for Human Space Exploration

**DOI:** 10.3389/fonc.2015.00273

**Published:** 2015-12-08

**Authors:** Livio Narici, Thomas Berger, Daniel Matthiä, Günther Reitz

**Affiliations:** ^1^Department of Physics University of Rome Tor Vergata and INFN-Roma2, Rome, Italy; ^2^Institute of Aerospace Medicine, German Aerospace Center (DLR), Cologne, Germany

**Keywords:** active radiation detectors, International Space Station, human space exploration, space radiation risk, database

## Abstract

A reliable radiation risk assessment in space is a mandatory step for the development of countermeasures and long-duration mission planning in human spaceflight. Research in radiobiology provides information about possible risks linked to radiation. In addition, for a meaningful risk evaluation, the radiation exposure has to be assessed to a sufficient level of accuracy. Consequently, both the radiation models predicting the risks and the measurements used to validate such models must have an equivalent precision. Corresponding measurements can be performed both with passive and active devices. The former is easier to handle, cheaper, lighter, and smaller but they measure neither the time dependence of the radiation environment nor some of the details useful for a comprehensive radiation risk assessment. Active detectors provide most of these details and have been extensively used in the International Space Station. To easily access such an amount of data, a single point access is becoming essential. This review presents an ongoing work on the development of a tool that allows obtaining information about all relevant measurements performed with active detectors providing reliable inputs for radiation model validation.

## Introduction

Space radiation risks (on humans and instrumentation) are possibly the most severe challenge posed to human exploration of deep space (1). A reliable space radiation risk assessment requires further understanding in radiation biology and also more details about the characteristics of the radiation.

Future mission planning will be based on radiation models which will have to be able to describe to a sufficient level of accuracy the radiation environment the crew will be living in. Such models are under development and consist of a combination of source models, transport models, and computer-aided design (CAD) ability to describe the habitat (either a spacecraft or a space base). Each part as well as the entire final model will have to be properly validated by measurements, and consequently, these measurements will have to provide a similar degree of detail as the model. Passive detectors cannot provide information about, for example, temporal and spatial evolution, or the primary kinetic energy of the ions. Often not even active detectors permit to measure all these parameters.

The construction of future space vessels and space bases is expected to be of similar complexity compared to the International Space Station (ISS). This makes the ISS an important “validation habitat” for the mentioned radiation models. The radiation impinging on the ISS is, however, modulated by the Earth magnetic field, and thus it has features that are not found in deep space. In particular, primary cosmic radiation at low latitude is heavily reduced comprising only high energy ions; charged particles with lower energies are deflected by the magnetic field. At high latitudes, this shielding effect of the magnetic field is much weaker and the radiation field is more similar to the one encountered in deep space.

Furthermore, in a region above Brazil (South Atlantic Anomaly, SAA), the tilt and shift of the magnetic dipole-axis compared to the rotation-axis of Earth result in a close approach of the radiation belt to the Earth’s surface. In the radiation belt, a large amount of low charge ions (mostly protons) of relatively low energy is trapped.

Due to the latitude-dependent magnetic shielding and the presence of the SAA active detectors are advantageous for model validation for deep space; they can be used to select measurements from specific geographical regions and permit to construct a proper dataset for validation, i.e., from high latitudes.

Ideally, measurements and model developments should proceed in a process defining both the most suitable areas in the ISS to perform experiments and the required parameters that are to be measured. Although this approach is usually not realized, a high number of measurements have been and are being performed, which are useful for model validation.

Teams working on radiation models and looking for data for validation often encounter difficulties as literature searches are not always fruitful. Sometime this is due to the difficulty to extract numerical values from articles or it can be due to the delay in the publication procedure. Also, these searches are most often quite time consuming. Therefore, researchers heavily rely on personal communications from teams they have ongoing ­collaborations with.

It is obvious that a far better approach is having a single access point where all data can be found. In the future, this should lead to a worldwide accessible database. In order to foster the development of such a database and to provide to the scientific community a simple tool for the fast and successful identification of suitable data, a *search book* is being created in which all relevant information is to be collected.

This review provides a description of this *search book* that is a soon to be web-published compilation of basic information on active detector measurements of radiation environment for human exploration risk assessment, in particular, when, where (in the ISS or also in or on other relevant satellites/spacecraft), how (what kind of active detector, what kind of measurement), and who (a contributor for each dataset to whom any question about the corresponding data might be addressed).

The goal is to provide this information interactively available on the web. As this work is ongoing the list of active detectors in this article is not complete, and it will be a continuing task to update the data.

## Time Frame and Measurements Considered

This review covers the ISS life span and includes also measurements performed during the same period at locations different than ISS, provided that they are of relevance for human space exploration. There are several reasons why the ISS is an ideal starting point:

(i) ISS is the best currently available test bed for radiation measurements aimed at model validation (see above);
(ii) detailed CAD simulations are being developed for ISS, and hopefully, these simulations will soon be able to track mass movements in the ISS; this would permit to compare radiation models and radiation measurements for different, well-defined shielding environments.
(iii) ISS is going to operate probably for another decade, providing the time frame needed for a coordinated approach of measurements and model development and validation (see above).

Concurrent measurements on Earth satellites as well as around Moon or Mars and in interplanetary space are also extremely important as they provide information about the radiation sources impinging on the ISS.

## The Summary Tables

Table 1 shows the measurement locations in the past 16 years, as well as the detector performing each measurement. A filled box indicates that measurements were taken during that year. Further details about the time and duration of each measurement as well as detector characteristics and relevant operational parameters will be provided on the web site.

**Table 1:** **Overview of active detector measurements since the beginning of the ISS era**.

Modulus	Detector	2000	2001	2002	2003	2004	2005	2006	2007	2008	2009	2010	2011	2012	2013	2014	2015
**Iss, internal**																	
USLab	Liulin-E094																
	IV-CPDS																
	ALTEA																
	Dosmap-Dostel																
Columbus	Dosis-Dostel-1																
	Dosis-Dostel-2																
	ALTEA																
	Tritel																
Zvezda	R-16																
	DB-8																
	Alteino																
	MTR-Dostel																
	Tritel																
Zarya	Alteino																
Pirs	Alteino																
	Liulin-5																
Russ Segment	Liulin-ISS																
MRM1	Liulin-5																
**Iss, external**																	
External-USLab	EV1-CPDS																
	EV2-CPDS																
	EV3-CPDS																
External-Columbus	R3DE																
	EuTEF-Dostel																
External-Zvezda	MTR-Dostel																
	R3DR																
**Satellite**	**Detector**	**2000**	**2001**	**2002**	**2003**	**2004**	**2005**	**2006**	**2007**	**2008**	**2009**	**2010**	**2011**	**2012**	**2013**	**2014**	**2015**
**Leo satellites**																	
Foton-M2	R3D-B2																
Foton-M3	Liulin-Photo																
Foton-M3	R3D-B3																
Foton-M4	RD3-B3																
Bion-M1	RD3-B3																
Resurs-DK1	PAMELA																
**Moon satellites**																	
Chandrayaan	RADOM																
LRO	CRaTER																
**Mars satellites**																	
Odyssey	MARIE																
**System**	**Detector**	**2000**	**2001**	**2002**	**2003**	**2004**	**2005**	**2006**	**2007**	**2008**	**2009**	**2010**	**2011**	**2012**	**2013**	**2014**	**2015**
**Mars cruise**																	
Curiosity	MSL-RAD																
**Mars surface**																	
Curiosity	MSL-RAD																

*Filled boxes indicate years in which measurements have been performed with the corresponding detector and on the corresponding location. To obtain more detailed information about the exact timing, the reader is suggested to read the relative references or contact the contact point (see Table 2). The table is still not complete (work in progress, see text)*.

We give a brief account of the latter in Table 2 together with an email address as contact point for each of the considered instruments. For any further information and details, the reader is addressed to the references.

**Table 2:** **Details of the active radiation detectors that have been active on the ISS or that have performed measurements relevant to human exploration**.

Detector	Sensor material	Year first use	No. of sensor	Typical sensor area and thickness		Telescope geometrical factor (single ended) (cm^2^ sr)	LET (Si) acceptance (keV/**μ**m)	Reference	Contact person
				Area (cm^2^)	Thickness (mm)				
R-16	Argon (ion. Chamb.)	2000	1	^a^	^a^	–	–	(2–9)	Victor V. Benghin, v_benghin@mail.ru
DB-8	Silicon	2001	1	1	0.3	–	–	(2–9)	Victor V. Benghin, v_benghin@mail.ru
Liulin^b^	Silicon	2001	1	2	0.3	–	–	(10–42)^c^	Tsvetan P. Dachev, tdachev@bas.bg
CPDS^d^	Silicon	2001	12	5.8, 9, 26.8	0.3–1.0–5.0	3.2	0.3–30	(44, 45)	Kerry T. Lee, kerry.t.lee@nasa.gov
Alteino	Striped Silicon	2002	8	64	0.4	23.8	0.3–430	(46–52)	Marco Casolino, casolino@roma2.infn.it
ALTEA	Striped Silicon^e^	2006	6	128	0.4	115	3–700	(55–74)	Livio Narici, narici@roma2.infn.it
Liulin-5	Silicon	2007	3	2.3	0.4	1	1.2–170	(75–82)^f^	Jordanka Semkova, jsemkova@stil.bas.bg
Dostel^g^	Silicon	2001	2	6.9	0.4	8.2	0.5–400	(16, 83–89)	Thomas Berger, Thomas.Berger@dlr.de
Tritel	Silicon^h^	2012	2	2.2	0.3	2.5	0.2–290	(90–95)	Hirn Attila, hirn.attila@energia.mta.hu
PAMELA	Striped Silicon^i^	2006	6	37.3	0.3	21.6	0.03–97	(96–112)	Roberta Sparvoli, sparvoli@roma2.infn.it
MARIE	Silicon	2002	8	5.8, 9, 26.8	0.3–1.0–5.0	3.2	0.5–57	(113–118)	Cary Zeitlin, cary.j.zeitlin@nasa.gov
CRaTER	Silicon^j^	2009	6	9.6	0.15–1.0	0.6–24.6^k^	0.2–2200	(119–126)	Nathan Schwadron, nschwadron@guero.sr.unh.edu
MSL-RAD	Silicon segmented^l^	2011	3	13.9, 2.3, 1.9^m^	0.3	0.17–0.72^k^	0.1–1000	(127–135)	Donald M. Hassler, hassler@boulder.swri.edu

*^a^Hemisphere connected with a cylinder, the internal radius of the hemisphere and the cylinder being 20 mm, the height of the cylinder being 20 mm*.

*^b^Several instruments in this family: *Liulin-E094, Liulin-ISS, Liulin-Photo, RADOM, R3DE, R3DR, R3D-B2, R3D-B3**.

*^c^See also Liulin-5 references*.

*^d^Four CPDS: one intra-vehicular (IV-CPDS) and three extra-vehicular (EV-CPDS)*.

*^e^Six identical telescopes deployed in different configurations: on an helmet-like holder, in a *X*, *Y*, and *Z* configuration, in a flat configurations*.

*^f^See also Liulin references*.

*^g^Several instruments in this family: DosMap-Dostel, MTR-Dostel, EuTEF-Dostel, Dosis-Dostel*.

*^h^Two sensors for each of the three cartesian directions (*X*, *Y*, *Z*) for a total of 6*.

*^i^PAMELA silicon tracker is part of a complex instrument featuring also a permanent magnet as well as a time of flight system (based on 6 fast plastic scintillators and a calorimeter. See references)*.

*^j^Each pair of sensors (thin–thick) is sandwiched on tissue equivalent plastic*.

*^k^Depending on the coincidence choice*.

*^l^RAD includes also a CsI(Tl) scintillator, a plastic scintillator, and an anti-coincidence system, see references*.

*^m^The top sensor is segmented into two rings, others are like the inner part of A, see references*.

*Further details in the references*.

## A Brief Description of the Detectors

The *R-16* detector (2–9) is a combination of two Argon filled ionization chamber with two different shielding. It has been also the first active detector in use in the ISS.

*The DB-8* (2–9) detector is similar to the Liulin (see below). Four DB-8 units are a part of the ISS radiation monitoring system (RMS). All the DB-8 units are identical and two independent sensors with different shielding operate in each of the DB-8 units. The DB-8 units were located in different locations in the ZVEDA module to provide measurements in different shielding environments.

*Liulin* (10–42) labels a set of small detectors of very similar dimensions and operational principles but differing in read out, storing, and other characteristics that tailored each detector to the experiments it was built for. Details on these instruments can be found in the references, e.g., Ref. (40). All Liulin-type instruments use silicon detectors and measure the deposited energy and the number of charged particles hitting the device, which can be converted to dose rate and particle flux. The first Liulin was developed in the late 80s (43) for use in the MIR Station. The ISS instruments are Liulin-E094 (April to August 2001), Liulin-ISS (September 2005 to June 2014), Liulin-5 (May 2007 to present), R3DE (February 2008 to September 2009), and R3DR (March 2009 to August 2010), the latter two have been deployed outside the ISS. Several Liulins (R3D-B2, R3D-B3, Liulin-Photo) have also been used in Foton and Bion satellites flying in Low Earth Orbit (LEO) and in the Chandrayaan-1 satellite around the moon (RADOM). The Liulin-5 (75–82) is the first of a new Liulin type: it is a telescope using three silicon detectors providing both a coincidence (telescope) and a non-coincidence read out (for direct dose measurements).

The charged particle directional spectrometer (*CPDS*) (44, 45) is a detector used by NASA to monitor ISS radiation environment. It is a telescope with a 12 element silicon stack with different thickness (6 thick, 5 mm and 6 thinner, 0.3 and 1 mm) and a Cerenkov detector at the bottom. Thick detectors are for particle identification. The individual sensor cards are identical in design to those used on the MARIE instrument (see below). Three CPDS have been deployed externally (EV-CPDS). EV1-CPDS and EV3-CPDS were aligned with the +*X* and −*X* axis in the ISS coordinate system, while EV2-CPSDS with the −*Z* axis. Another detector (IV-CPDS) was deployed inside the USLab initially pointing to the +*X* direction and since 8 August 2006 to the +*Z* direction.

*Alteino* (46–52), often referred to as SilEye3, is an upgraded SilEye (53), which was successfully deployed in the MIR station. As all the ALTEA systems it originates from investigations of the radiation potentially causing the “Light Flash” anomalous perception to the astronauts [Ref. (54) and references therein]. Alteino features a stack of eight silicon striped sensors (each 80 mm × 80 mm × 0.38 mm) and two plastic scintillators: one in front of and one behind the silicon stack. Sensors are segmented in alternating directions (*X* and *Y*). The direction and the height in the stack of eight provide a set of three coordinates for each sensor traversal and for each particle, and permit to track its direction. Alteino is a pure telescope recording radiation only when both scintillators are hit. The detector has the size of a shoebox, and it is able to measure the LET in silicon and the flux and the trajectory of each ion, using the ability to know which of the sensor strip has been hit. Alteino was deployed in the Russian segment of the ISS, in the Russian space program and then in an ESA sponsored experiments under the ALTCRISS project. Alteino data were downloaded periodically via PCMCIA cards. Alteino provided detailed particle spectra in the Russian modules.

*ALTEA* (55–74) is a system of six identical silicon telescopes. Each telescope is similar to Alteino (also in size) but lacking the scintillators and featuring six striped silicon sensors with twice the area of the sensors used in Alteino (each 160 mm × 80 mm × 0.38 mm). The bi-directional geometrical factor is 230 cm^2^ sr and the system is auto-triggered by traversing ions. The six telescopes are identical and in the periods of interest have been deployed either on a helmet shaped holder in different locations in the USLab, on a three axis (*X*, *Y*, and *Z*) holder in other locations in the USLab or in a flat configuration for measuring effectiveness of shielding materials in one location in Columbus. ALTEA data are downlinked via real time telemetry. A software package on ground provides flux, dose, dose equivalent, and spectra in real time. ALTEA ISS surveys allowed for the 3D characterization of the radiation environment in the USLab (relevant for model validation). Also of relevance is the study of the iron abundance (ISS – USLab), which is apparently lower than expected.

*DosTel* (16, 83–89) is a detector family based on two silicon detectors, each with an area of 6.94 cm^2^ arranged in telescope geometry. With this setup, the DosTel applied for various experiments onboard space stations and shuttle missions can measure energy deposition of radiation hitting a detector (“dose mode”) or coincidental hits in the two detectors (“telescope” or “LET” mode). From 2009 onward, two identical DosTel units have been deployed in Columbus looking in two directions (*X* and *Y*). The long duration of the DosTel measurements in Columbus permits to study the variation of the radiation during a long solar modulation period.

*TRITEL* (90–95) is a set of three small two elements silicon telescopes, mounted in a 3D configuration (*X*, *Y*, and *Z*). Each telescope is made of two sensors, 220 mm × 0.3 mm, and has a geometrical factor of 5.1 cm^2^ sr. All sensors are identical, fully depleted, passivated implanted planar silicon (PIPS) detectors. TRITEL has been deployed in the ISS since 2012. TRITEL has been deployed in the Columbus modulus in the EPM rack (TRITEL-SURE, close to DosTel) and in the Zvezda modulus (TRITEL-RS).

*PAMELA* (96–112) was developed from an instrument that flew aboard the balloon missions MASS, TS93, and CAPRICE, with a design optimized for the study of antimatter in the cosmic radiation. For this type of investigation, it is necessary to have information about the particle charge, energy, and type of interaction from several redundant sub-detectors, in order to uniquely identify rare particles from background. It is composed of a Time-of-Flight (ToF) system, a magnetic spectrometer (MS), a sampling electromagnetic calorimeter (SeC), and a neutron detector (ND).

The ToF comprises six layers of fast plastic scintillators arranged in three double planes (S1, S2, and S3), with alternate layers placed orthogonal to each other. ToF information for charged particles is combined with track length information from the MS to determine particle velocities; particle charge (*Z*) can be determined up to *Z* = 8.

The MS consists of a permanent magnet and a silicon tracker (six equidistant 300 μm thick silicon detector planes inserted inside the magnetic cavity). Ionization loss measurements are also made in the silicon planes.

The SeC is made of 44 single-sided silicon sensor layers (380 μm thick) interleaved with 22 plates of tungsten absorber. The main task of the calorimeter is to select positrons and antiprotons from like-charged backgrounds, and it is therefore not of interest in our case; however, it also provides a measurement of the energy of the incident electrons independent from the MS, thus allowing a cross-calibration of the two energy determinations.

The ND is placed below the calorimeter with the aim to increase the electromagnetic and hadronic discrimination capability of the Pamela instrument.

PAMELA’s most important scientific results are related to the anomalous positron abundance. Here, however, the highest interest is in the extraordinary spectrometric ability in the low energy regime (below few GeV/n) for small *Z* (*Z* < 8) ions.

*MARIE* (113–118) is a telescope with an eight element silicon stack with Cerenkov detector at the bottom with different thickness (4 thick, 5 mm and 4 thinner, 0.3 and 1 mm). Thick detectors are for particle identification. The individual sensor cards are identical in design to those used on the CPDS instrument (see above). MARIE has been mounted on Odyssey’s equipment deck, pointing opposite to the spacecraft’s velocity vector. Odyssey is in a circular 2-h polar orbit around Mars. Forward field of view (FOV) points into deep space, rear FOV is partially blocked by Mars. MARIE performed the first characterization of the Martian radiation environment aimed at the risk assessment for human exploration.

*CRaTER* (119–126) is a telescope with three silicon detector pairs sandwiching pieces of tissue-equivalent plastic. Each pair has one thin detector (150 mm) for measuring high-LET particles and one thick detector (1 mm) for low-LET particles. CRaTER is part of LRO in orbit around the Moon. In nominal orbit, one end of telescope points zenith, the other nadir (toward lunar surface) with the rear FOV entirely filled by the lunar disk. LRO was in a circular 2-h polar orbit above the Moon for the prime mission and is now in an elliptical orbit. The overall dimensions are 24 cm × 23 cm × 16 cm. Of important is the long period of observations that permits to investigate the evolution of the deep space radiation environment during the solar cycle.

*RAD* (127–135) is a three-element silicon stack, a CsI(Tl) scintillator with p-i-n diode readout and a plastic scintillator at the bottom of the stack; an anti-coincidence system surrounds the CsI(Tl) scintillator and the plastic scintillator and enables their use as neutral particle detectors. It is mounted on the top deck of Curiosity rover pointing to the zenith. The rover is controlled so that tilt does not exceed 10°. During cruise phase, RAD was shielded from above by the Descent Vehicle and from below by the heat shield. On Mars, RAD was shielded by Martian atmosphere (~20 g cm^−2^ CO_2_). The detector is small (mass 1.56 kg, total volume 240 cm^3^) and has several coincidence and anti-coincidence capabilities. RAD allowed for the first detailed measurements of the Mars surface radiation environment.

## Active Detectors in Space: The Future

With the advances of the field, active detectors may partly replace passive detectors. Power requirements as well as required sensor dimensions are slowly entering the region of acceptability for a widespread use in space, both as area detectors (for which constraints on dimensions and power are not so strong and therefore requirements for detailed measurement of the radiation field can be fulfilled) and as personal detectors. In the latter case, the miniaturization would not allow for a complete characterization of the radiation field. Nevertheless, active personal dosimeters certainly would provide a more comprehensive picture than passive detectors, and they would permit real time monitoring and alarming capabilities [see, for example, Ref. (136)]. It would be desirable that the data of all future active devices flow into a network of databases in order to make it available in quasi real time for all interested teams.

## Conclusion

The development of a *search book* comprising information about radiation measurements relevant for the validation of models for human exploration has been reported. While not yet complete the extent of measurements of active detectors is already impressive and constitutes a valuable tool for anyone developing or validating radiation models for deep space spacecraft or habitats.

Complementing information about other measurements and detectors will be added in the future, and a similar approach for passive detectors should be started. A final output on a web page is foreseen.

## Author Contributions

All authors (LN, TB, DM, and GR) have participated to the idea and to the writing of the Search Book and of this first mini review.

## Conflict of Interest Statement

The authors declare that the research was conducted in the absence of any commercial or financial relationships that could be construed as a potential conflict of interest.
